# Worms avoid a cat sensed repellent

**DOI:** 10.17912/micropub.biology.000331

**Published:** 2020-11-28

**Authors:** Brianna Ramos, Gareth Harris

**Affiliations:** 1 1 University Drive, California State University Channel Islands, Camarillo, Ca, 93012

**Figure 1. C. elegans avoid a cat sensed repellent:**
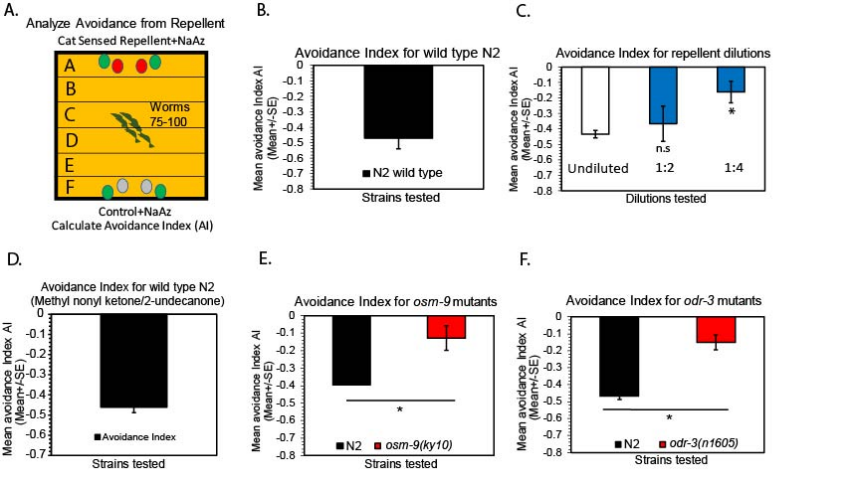
**A-F** A) Schematic of behavioral assay to examine avoidance behavior. Wild type worms were examined for avoidance to cat sensed repellents using a population avoidance assay (See methods), B) Wild type N2 worms avoid a cat sensed repellent, Boundary, (n=6), C) Wild type N2 worm avoidance to different repulsive odor concentrations of Boundary. Wild type N2 worms were examined in response to varying dilutions of cat sensed repellent Boundary, 1:2, 1:4 dilutions and undiluted odor cues used, (n=3), D) Wild type N2 worms were examined in response to undiluted methyl nonyl ketone (2-undecanone), (n=8), E) Wild type worm repulsion from cat sensed repellent requires distinct sensory transduction molecules. *osm-9* was identified to mediate repulsion to the cat sensed repellent (Boundary) based on avoidance behavior examined in *osm-9* mutants. *osm-9(ky10)* mutants, (n=3), F) *odr-3,* which encodes a G-protein is required for avoidance behavior to the cat repellent (Boundary). *odr-3*(*n1605)* mutants examined for avoidance is shown, (n=3), For all data analysis, a *Student’s* t-test was performed when comparing wild type hermaphrodite worms to all mutant hermaphrodite worms tested on the same day in parallel conditions. Mean ± SEM, *Student’s* t-test, * p ≤ 0.05, p ≤ 0.01**, p ≤ 0.001***. For all assays, n=number of days tested.

## Description

An organism’s behavior that promotes various behavioral and physiological responses can be influenced by olfactory behavior. Animals across the phyla constantly utilize chemosensory functions in order for survival (Lessing and Carlson, 1999, Ache and Young, 2005, Chaisson and Hallem, 2012). Organisms are also able to couple odor sensation with physiological responses and behavioral states to coordinate specific behavioral responses involved in bonding, social interaction, mating and feeding. For example, mammals, such as cats respond to odors using olfaction and respond based on processing of these types of cues at multiple levels of the brain to coordinate behavior (Hart *et al.*., 1985, Miyazaki *et al.*., 2017, Jacinto *et al.*., 2018). Despite understanding these important strategies, the neural molecules and circuit’s underlying these behaviors are not fully understood. Cats respond to olfactory cues, including repulsive chemicals that drive avoidance behavior, such as, methyl nonyl ketone and others (Wolski *et al.*., 1984). One repulsive spray that drives repulsion in many cats is Boundary. This cat repellent has been shown to produce an aversive response in cats.

We use the invertebrate nematode, *Caeanorhabditis elegans* to understand behavioral responses to mammalian sensed cues. *C. elegans* avoid a number of chemical stimuli, including, 1-octanol, 2-nonanone, carvone and methyl salicylate, among others (Troemel *et al.*., 1995, Treomel *et al.*., 1997, Luo *et al.*., 2015, Ellington *et al.*., 2020). We examined any chemosensory response to this cat repellent in adult *C. elegans* hermaphrodites and begin to characterize the mechanisms underlying these chemosensory responses. We have provided a platform to potentially study repulsive behavior to mammalian sensed odor cues based on our observations in these experiments. Using a chemotaxis assay, we have examined how wild type N2 worms behave when exposed to the cat sensed repulsive spray, Boundary (Bargmann **et al.*,*1991, Bargmann and Horvitz, 1993, Troemel **et al.*,*1997). We have taken advantage of this behavior paradigm to begin dissecting the chemosensory/neural circuit mechanisms that allow coordinated sensory-dependent behavior when exposed to this cat sensed odor cue.

The odors present in Boundary (cat repellent-contains 2-undecanone/methyl nonyl ketone and other chemicals) have been shown to be a strong repellent to many cats (Wolski *et al.*., 1984, Bohbot *et al.*., 2011). We examined whether *C. elegans* show any attraction or avoidance to this cat sensed repellent using a population avoidance assay (as described in Troemel *et al.*., 1997; Fig. 1A). We examined wild type young N2 adults in the presence of undiluted chemical repellent, Boundary, and analyzed chemotaxis behavior over 0-60 minutes after the cat repellent cue was added to the avoidance assay plate (Fig. 1A, Schematic Diagram of assay). Interestingly, upon examining wild type adults, we found repulsion was evident over a 60 minute period after the odor cues were added to the plate (Avoidance Index: ~0.5 index, Fig. 1A-1B). To further characterize the response, we also examined wild type animals at diluted concentrations of the repellent, including, undiluted tested against 1:2 and 1:4 dilutions (Fig. 1C). These concentrations were compared to undiluted repellent each time using wild type N2 worms (Fig. 1C). Dilutions of repellent resulted in some response at 1:2 dilutions, and significantly reduced response at 1:4 dilutions across 60 minutes (Fig. 1C). Taken together, these data suggest that we have identified a known cat repellent, that is also repulsive to wild type *C. elegans* adults (See Fig. 1A-C). We also examined one of the chemicals present in Boundary, ‘2-undecanone’ (methyl-nonyl ketone, Sigma Aldrich) for repulsion behavior to confirm the repulsive nature of this cat sensed cue. Methyl nonyl ketone (2-undecanone) has been previously shown to be an animal and insect repellent (Innocent *et al.*., 2008). Interestingly, this is also repulsive to wild type worms, as wild type N2 worms avoided this cue (Fig. 1D). Suggesting, Boundary and one of its chemical constituents, methyl nonyl ketone (2-undecanone) are repulsive to wild type *C. elegans*.

We sought to identify the molecular mechanisms that mediate this repulsive response to a cat sensed cue. Through the examination of sensory signals required for *C. elegans* odor avoidance, we identified ion channels and G-proteins expressed in worm sensory neurons to be involved in avoidance to the cat repellent, Boundary. Specifically, we have confirmed *osm-9-*encoding TRP channels to be important for repulsion to Boundary, based on *osm-9* mutants showing defective repulsion behavior to Boundary (Fig. 1E). *osm-9(ky10)* mutants are defective across 60 minutes using the repellent assay (Troemel *et al.*., 1997, Colbert *et al.*., 1997, Tobin **et al.*,* 2002, Hiliard *et al.*., 2004, Kahn-Kirby *et al.*., 2004, Fig. 1E). Suggesting, *osm-9-*dependent signaling is required for repulsion responses to this cat sensed repellent. We also examined mutants lacking other sensory transduction genes, including, G-alpha protein subunits that are primarily expressed in amphidal sensory neurons (Jaansen *et al.*., 1999). ODR-3 G-alpha protein subunit is also required based on *odr-3(n1605)* mutantsshowing defective avoidance behavior. *odr-3* has also been previously implicated in attraction and avoidance behaviors (Fig. 1F, Roayaie *et al.*., 1998, Harris *et al.*., 2014). Overall, we show wild type worms avoid this cat sensed repellent and 2-undecanone (methyl nonyl ketone). Therefore, providing a platform for further investigation of the molecular mechanisms and neural circuitry required for aversion to mammalian sensed cues.

## Methods

Wild type worms were reared as previously described (Brenner *et al.*., 1974). Wild type worms were cultivated on *E. coli* OP50 standard lab food source prior to testing in avoidance behavioral assay. Wild type (N2) Bristol worms (CGC, Minnesota) were used in all avoidance assays and compared to all mutants tested in parallel. 10 cm square plates (Thermofisher) were used for behavioral assays with no food present on the assay plate (Troemel *et al.*., 1997). All wild type and mutant animals were grown at 20-23 degrees with sufficient *E.coli* OP50 food source prior to examination of all worms. All worms tested in this present study were 1 day old adult hermaphrodite animals under non-starved conditions.

**Assay to measure avoidance to known cat sensed repellents**

To examine the avoidance of the cat sensed odor repellent (Boundary-contains methyl nonyl ketone/2-undecanone and other chemicals, Petco), chemotactic avoidance assays were performed essentially as previously described in Troemel *et al.*., 1997. Worms were washed 3 times with S. basal prior to square plate assay placement in order to more thoroughly remove food contaminants. Briefly, animals were placed in the center of a square plate that was divided into sectors A – F and 2 drops of 1 µl of Boundary was added to one side and 2 drops of 1 µl S. basal was added to the opposite side of the plate as a control. Then, 2 drops of sodium azide (NaAz) was placed on the outer sides of the stimuli and control in order to obtain an accurate count of the worms. Approximately 100 – 150 young adult worms were used in each avoidance assay. Avoidance behavior was analyzed by counting the number of worms in the sectors A-B, C-D and E-F with E-F being furthest away from the repellent odor sources (Fig. 1). All worms were examined and counted at 60 minutes after odor addition to the assay plate. The avoidance index was calculated as the number of animals in sectors A and B(repellent) minus the number of animals in the sectors E and F(Control) and normalized with the total number of animals in all 6 sectors on the plate. For assays that examine dilutions of cat sensed repellent cue, repellent was diluted in S. basal to achieve 1:2 and 1:4 dilutions. All mutant animals were compared to wild type animals tested in parallel on the same day. For assays that examine 2-undecanone/methyl nonyl ketone avoidance (Sigma Aldrich), the assay used identical conditions. Mean+/-SE, *Students* t-test was performed as statistics used for examination of differences between each mutant and wild type animals tested on the same day under identical conditions.

## Reagents

**Strains tested and shown in this study**

Wild type (N2), CX10 *osm-9(ky10)IV*, CX3222 *odr-3(n1605)V*. All strains were provided by the CGC(*Caenorhabditis* Genetics Center) at the University of Minnesota, which is funded by NIH Office of Research Infrastructure Programs (P40 OD010440).

**Reagents used for this study:**

1) Boundary (consisting of methyl nonyl ketone/2-undecanone and other chemicals), was purchased from Petco.com, 2) 2-undecanone (methyl nonyl ketone), was purchased from Sigma Aldrich.
